# A systematic review of associations between common *SNCA* variants and clinical heterogeneity in Parkinson’s disease

**DOI:** 10.1038/s41531-021-00196-5

**Published:** 2021-07-01

**Authors:** Camilla Christina Pedersen, Johannes Lange, Marthe Gurine Gunnarsdatter Førland, Angus D. Macleod, Guido Alves, Jodi Maple-Grødem

**Affiliations:** 1 The Norwegian Centre for Movement Disorders, Stavanger University Hospital, Stavanger, Norway; 2 Department of Chemistry, Bioscience and Environmental Engineering, University of Stavanger, Stavanger, Norway; 3 Institute of Applied Health Sciences, University of Aberdeen, Aberdeen, UK; 4 Department of Neurology, Stavanger University Hospital, Stavanger, Norway

**Keywords:** Parkinson's disease, Genetics

## Abstract

There is great heterogeneity in both the clinical presentation and rate of disease progression among patients with Parkinson’s disease (PD). This can pose prognostic difficulties in a clinical setting, and a greater understanding of the risk factors that contribute to modify disease course is of clear importance for optimizing patient care and clinical trial design. Genetic variants in *SNCA* are an established risk factor for PD and are candidates to modify disease presentation and progression. This systematic review aimed to summarize all available primary research reporting the association of *SNCA* polymorphisms with features of PD. We systematically searched PubMed and Web of Science, from inception to 1 June 2020, for studies evaluating the association of common *SNCA* variants with age at onset (AAO) or any clinical feature attributed to PD in patients with idiopathic PD. Fifty-eight studies were included in the review that investigated the association between *SNCA* polymorphisms and a broad range of outcomes, including motor and cognitive impairment, sleep disorders, mental health, hyposmia, or AAO. The most reproducible findings were with the REP1 polymorphism or rs356219 and an earlier AAO, but no clear associations were identified with an *SNCA* polymorphism and any individual clinical outcome. The results of this comprehensive summary suggest that, while there is evidence that genetic variance in the *SNCA* region may have a small impact on clinical outcomes in PD, the mechanisms underlying the association of *SNCA* polymorphisms with PD risk may not be a major factor driving clinical heterogeneity in PD.

## Introduction

There is great heterogeneity in both the clinical presentation and the rate of disease progression between patients with Parkinson’s disease (PD)^1,2^. While PD is characterized by motor symptoms such as resting tremor, bradykinesia, rigidity, and postural instability, patients also experience a variety of non-motor symptoms, including cognitive impairment and dementia, sleep disorders, depression, anxiety, hallucinations, and hyposmia^3^. These non-motor symptoms complicate the clinical management of the disorder and are significant determinants of poor quality of life for patients and their caregivers. The observed heterogeneity can also pose prognostic difficulties in a clinical setting, and a greater understanding of the risk factors that contribute to modify disease course is of importance in terms of informing patients more accurately how they may be affected by the disease and developing personalized medicine strategies for patient management. Further, there is increased interest in targeting patients with a higher risk of specific disease outcomes in tailored clinical trials.

A logical first step in seeking genetic modifiers for progression in PD is to examine well-established PD susceptibility genes. These include *SNCA*, the gene encoding the α-synuclein protein that constitutes the major protein component of Lewy bodies (LBs)^4^. The abnormal aggregation of α-synuclein has a primary role in the formation of the LBs and other α-synuclein pathological aggregates and is regarded as a critical step in the molecular pathogenesis of PD^5^. In familial cases of PD, specific mutations or copy number variations (CNVs) of *SNCA* have been shown to cause increased production of α-synuclein and this is correlated with increasing disease severity^6,7^. Such mutations and CNVs of *SNCA* are rare in the general PD population, but candidate gene studies and genome-wide association studies (GWAS) have established the *SNCA* locus as a risk factor for idiopathic PD^8,9^ and accordingly as a promising candidate for disease modification.

The analysis of common polymorphisms in the *SNCA* region with clinical outcomes or age at PD onset has received much attention, but the use of different clinical scales, populations, and study designs complicates the comparison of the results, and no clear disease-modifying polymorphisms have been identified so far. For a better understanding of the impact of *SNCA* genetic polymorphisms on the progression of disease, a comprehensive overview of the associations between clinical evaluations and *SNCA* genotypes in PD is necessary. This systematic review aims to summarize and compare all available primary literature that has evaluated the association between *SNCA* polymorphisms and any PD clinical outcome. The review is limited to the analysis of patients with idiopathic PD, but given the broad symptomatology accredited to PD, no restrictions were placed on the clinical outcome measures evaluated in the studies. The potential contribution of *SNCA* polymorphisms to the clinical manifestations of PD is summarized. Further, the implications of the results for our understanding on the pathophysiology of PD and the design of future studies are discussed.

## Results

### Characteristics of included studies

**Fig. 1:**
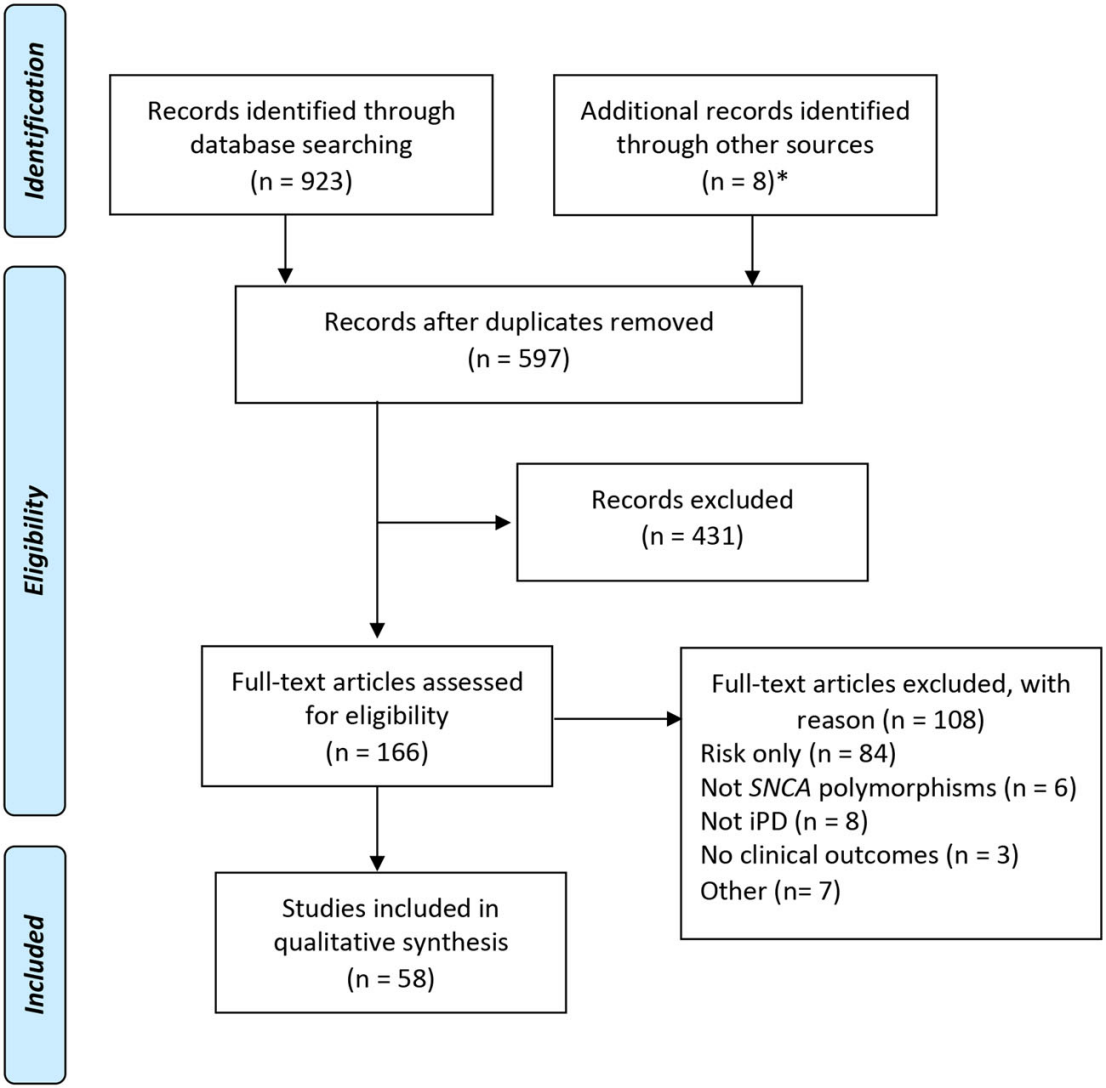
Study selection flow diagram. Asterisk (*): eight further articles were retrieved from a manual search of the 50 evaluated papers’ references (*n* = 5) and from the wider PD literature (*n* = 3). iPD idiopathic Parkinson’s disease, SNP single-nucleotide polymorphism.

The search strategy yielded 597 unique studies (Fig. 1) of which 58 were found to be eligible for inclusion. Of the 58 studies included in the review, 24 evaluated PD age at onset (AAO; median size 832, range 81–28,568 participants) and 41 evaluated clinical features of PD (median size 330, range 50–2011 participants), and predominantly included patients of European (Europe, Australia, or North America; *n* = 37) or Asian ancestry (*n* = 24). Only one study population was from South America (Brazil). All studies used defined diagnostic criteria for the diagnosis of PD (Table 1) as specified in the inclusion criteria for the review, though notably one study additionally incorporated data from a commercial genotyping project (23andMe) in which the diagnosis of PD was self-reported^10^. Assessment of bias in each study revealed that those that stood out as having the lowest number of sources of potential bias replicated the findings in independent cohorts, properly accounted for multiple testing, or were based on population-based cohorts (Supplementary Table 1).

**Table 1:** Overview of studies included in the review.

First author	Region^a^	Cohort or main site^b^	Diagnosis^c^	PD, *n*^d^	Age at onset or diagnosis^e^	Age at examination^e^	Disease duration at examination^e^	PD in longitudinal study, *n*	Follow-up, years^f^
Kim, 2020^26^ ^g^	UK	CamPaIGN	UKBB	141	70.2 ± 9.6			124	7.8 ± 3.5
Krohn, 2020^48^	Europe, North America	Multicenter	UKBB and MDS	1013					
Stoker, 2020^65^	UK	CamPaIGN	UKBB	142				85^h^	Max 18 years
Blauwendraat, 2019^10^	Global	IPDGC & 23andMe	UKBB	17,996 (IPDGC); 10,572 (23andMe)	62.14 ± 12.08 (IPDGC); 60.71 ± 9.98 (23andMe)				
Fernandez-Santiago, 2019^24^	Spain	Hospital Clínic de Barcelona & IPDGC	UKBB	898 (Spanish); 4170 (IPDGC)					
Luo, 2019^37^	China	Shanghai Ruijin Hospital	UKBB	50		64.6 ± 5.4		50	30 months
Ng, 2019^34^ ^g^	Singapore	National Neuroscience Institute	Gelb	172					
Si, 2019^27^	China	First Affiliated Hospital of Nanjing Medical University	UKBB	62					
Zhang, 2019^66^	China	Xiangya Hospital, Central South University	UKBB	81		53.76 ± 10.72			
Zheng, 2019^39^	China	West China Hospital	UKBB	291		61.8 ± 11.4			
Bjørnarå, 2018^47^	Global	Oslo University Hospital, Drammen Hospital & PPMI	UKBB & Mixed (PPMI)	325 (Norway); 382 (PPMI)		64.9 ± 9.4 (Norway)	9.2 ± 6.9 (Norway)		
Corrado, 2018^18^	Italy	Multicenter	Gelb	426	Median 62 (IQR 55–68)	Median 74.0 (IQR 68–79)	Median 11.0 (IQR 8–14)	426	11.3
Sampedro, 2018^67^	Global	PPMI	Mixed	112		60.2 ± 9.5	6.7 ± 6.9 months		
Shu, 2018^28^	China	Xiangya Hospital of Central South University	UKBB	724					
Campelo, 2017^35^	Brazil	Onofre Lopes University Hospital	UKBB	105	55.7 ± 11.9	64.42 ± 11.69	8.80 ± 5.78		
Caspell-Garcia, 2017^68^ ^g^	Global	PPMI	Mixed	423		61.7 ± 9.7	6.7 ± 6.5 months		
Cooper, 2017^29^	USA	UPenn & PANUC	UKBB	251 (UPenn); 559 (PANUC)		Median 71, IQR 64–76 (UPenn); median 67, IQR 62–74 (PANUC)	Median 7, IQR 4–11 (UPenn); median 8, IQR 4.5–12 (PANUC)	230	4 (range 1–7)
Huertas, 2017^43^	Spain	Virgen del Rocío Hospital	UKBB	298	55 ± 13		6 ± 6	298	11
Li, 2017^49^	China	Shanghai Ruijin Hospital	UKBB	152	Range 50–80				
Toffoli, 2017^50^	Italy	Multicenter	UKBB	57	67.9 ± 7.0	71.6 ± 5.7			
Zheng, 2017^30^	China	West China Hospital	UKBB	258					
Zheng, 2017^69^	China	West China Hospital	UKBB	330		61.9 ± 11.1			
Cheng, 2016^70^	China	West China Hospital	UKBB	1053	52.23 ± 10.65	56.84 ± 10.58	4.62 ± 4.02		
Dan, 2016^44^	China	CNCPD	UKBB	1047	57.34 ± 10.62	61.98 ± 10.15	4.60 ± 4.01		
Davis, 2016^22^	Global	UW & PPMI	UKBB	418 (UW); 368 (PPMI)	60.4 ± 11.1 (UW); 61.4 ± 9.9 (PPMI)			425	^i^
Shi, 2016^36^	China	CNCPD	UKBB	2011	58.6 ± 10.4	62.7 ± 10.1			
Wang, 2016^31^	China	Shanghai Ruijin Hospital	UKBB	296	57.87 (10.04)	62.60 (9.40)	4.74 (4.18)	22	4
Chen, 2015^71^	China	West China Hospital	UKBB	1276	56.32 ± 11.52	60.45 ± 11.52	4.53 ± 4.15		
Chen, 2015^53^	China	Shanghai Ruijin Hospital	UKBB	218	60.6 ± 7.4	64.4 ± 6.8	3.7 ± 2.9		
Huang, 2015^19^	Australia, China	Australian PD Research Network & Ruijin Hospital	UKBB	Australia (123); China (289)	60 ± 11 (Australia); 58 ± 10 (China)	68 ± 9 (Australia); 63 ± 9 (China)			
Chung, 2014^21^	Global	GEO-PD	Mixed	6012	58.2 ± 11.6	66.5 ± 10.6	Median 6 (range 0–54)		
Guo, 2014^72^	China	West China Hospital of Sichuan University	UKBB	1011	56.67 ± 11.87	60.51 ± 11.60	4.41 ± 4.07		
Markopoulou, 2014^38^	USA	MEPD	Bower	922	Median 62.2 (range 23.3–88.0)	Median 68.0 (range 30.8–91.4)	Median 3.3	922	Median 7.8 (range 3.3–13)
Mata, 2014^40^	USA	Multicenter	UKBB	1079	62.2 ± 8.7	68.8 ± 9.1	6.6 ± 5.4		
Yarnall, 2014^73^	UK	ICICLE-PD	UKBB	219		65.9 ± 9.7	5.5 ± 5.0		
Brockman, 2013^23^	Germany	University of Tübingen	UKBB	1396	56.91 ± 11.91				
Li, 2013^25^	China	West China Hospital	UKBB	685	54.55 ± 10.48				
Williams-Gray, 2013^41^ ^g^	UK	CamPaIGN	UKBB	142	70.2 ± 9.6			142	7.2 ± 2.8
Cardo, 2012^74^	Spain	Multicenter	UKBB	1169	59 ± 12				
Ritz, 2012^32^	USA	PEG	UKBB	363				232	5.1 ± 2.2
Chung, 2011^11^	USA	Mayo Clinic	Bower	1103	Median 62.2 (range: 23.3–88.0)	Median 68.0 (range: 30.8–91.4)			
Ding, 2011^75^	USA	HBS	UKBB	375	61.00 ± 11.51	66.42 ± 10.83			
Elbaz, 2011^76^	Global	GEO-PD	Mixed	5302					
Factor, 2011^45^	USA	NGRC	UKBB	500		67.7 ± 10.8	8.5 ± 6.2		
Factor, 2011^77^	USA	NGRC	UKBB	499		67.7 ± 10.8	8.5 ± 6.3		
Huang, 2011^33^ ^g^	Australia	Sydney	UKBB	123	60 ± 11	68 ± 9	8 ± 7		
Kim, 2010^78^	South Korea	Seoul National University Hospital	UKBB	878	56.6 ± 9.5	64.2 ± 9.0			
Yu, 2010^79^	China	West China Hospital & First Affiliated Hospital of Sun Yatsen University	UKBB	332	54.37 ± 11.19	58.24 ± 11.21			
De Marco, 2008^80^	Italy	University of Catanzaro & Misericordia Hospital	UKBB	228					
Kay, 2008^12^	USA	NGRC	UKBB	1802	58.6 ± 11.7	68.0 ± 10.6			
Verbaan, 2008^54^	Netherlands	PROPARK	UKBB	295	48.4 ± 11.2	60.2 ± 10.6	11.8 ± 6.3		
Goris, 2007^42^	UK	CamPaIGN & multicenter	UKBB	659	63 (range 25–91)	71 ± 10		109	3.5
Ross, 2007^13^	Ireland	Unspecified	Gelb	186	50 ± 11	61 ± 12			
Winkler, 2007^14^	Germany, Serbia	Unspecified	UKBB	397	47 ± 11	55 ± 11			
Hadjigeorgiou, 2006^20^ ^g^	Greece	Larissa University Hospital	Bower	178	63.3 ± 9.6	69.5 ± 9.7			
Maraganore, 2006^15^	Global	GEO-PD	Mixed	2692	Range 17–88	Range 21–99			
Tan, 2003^17^ ^g^	Singapore	Singapore General Hospital	UKBB	206					
Tan, 2000^16^ ^g^	Singapore, Germany	Singapore General Hospital & unspecified	Gelb	100	53.4 ± 11.5	61 ± 12			

^a^Countries/regions from which the patients were recruited. If >2 continents were included, global is indicated.

^b^The study site or study name, when reported. If >2 study sites were included in each cohort, “multicenter” is indicated.

^c^The criteria by which PD was diagnosed. Where “mixed” is indicated for multicenter studies, GEO-PD (included in Chung et al.^21^, Elbaz et al.^76^, and Maraganore et al.^15^) used UKBB, Gelb, Bower, the Core Assessment Program for Intracerebral Transplantations (CAPIT) and criteria described in Pals et al.^81^ and PPMI (included in Bjørnarå et al.^47^, Sampedro et al.^67^, and Caspell-Garcia et al.^68^) used criteria outlined in the study protocol (http://www.ppmi-info.org; Parkinson Progression Marker Initiative^82^).

^d^The number of patients with PD included in the whole study cohort. In some studies, genetic data or longitudinal follow-up data are only available for a subset of patients.

^e^Mean ± SD in years (unless otherwise stated) given when reported for all PD patients in the cohort.

^f^Follow-up time included both prospective and retrospective studies. Mean ± SD in years (unless otherwise stated).

^g^These articles were retrieved from a manual search of the 50 evaluated papers’ references (*n* = 5) and from the wider PD literature (*n* = 3).

^h^Number of idiopathic PD in the analysis.

^i^Follow-up time: cases with at least three scores measured over a minimum of 1 year were included.

*CamPaIGN* Cambridgeshire Parkinson’s Incidence from GP to Neurologist, *CNCPD* Chinese National Consortium on Neurodegenerative Diseases, *GEO-PD* Genetics and Epidemiology of PD, *HBS* Harvard Biomarker Study, *ICICLE-PD* Incidence of Cognitive Impairment in Cohorts with Longitudinal Evaluation-PD, *IPDGC* International Parkinson Disease Genomics Consortium, *MDS* International Parkinson Disease and Movement Disorder Society, *MEPD* Molecular Epidemiology of Parkinson’s Disease, *NGRC* NeuroGenetics Research Consortium, *PANUC* Pacific Northwest Udall Center, *PD* Parkinson’s disease, *PEG* Parkinson Environment Gene, *PPMI* Parkinson’s Progression Markers Initiative, *PROGENI* Parkinson’s Research: The Organized Genetics Initiative, *PROPARK* profiling Parkinson’s disease, *UKBB* UK Parkinson’s Disease Society Brain Bank, *Upenn* University of Pennsylvania Cohort, *UW* University of Washington.

**Fig. 2:**
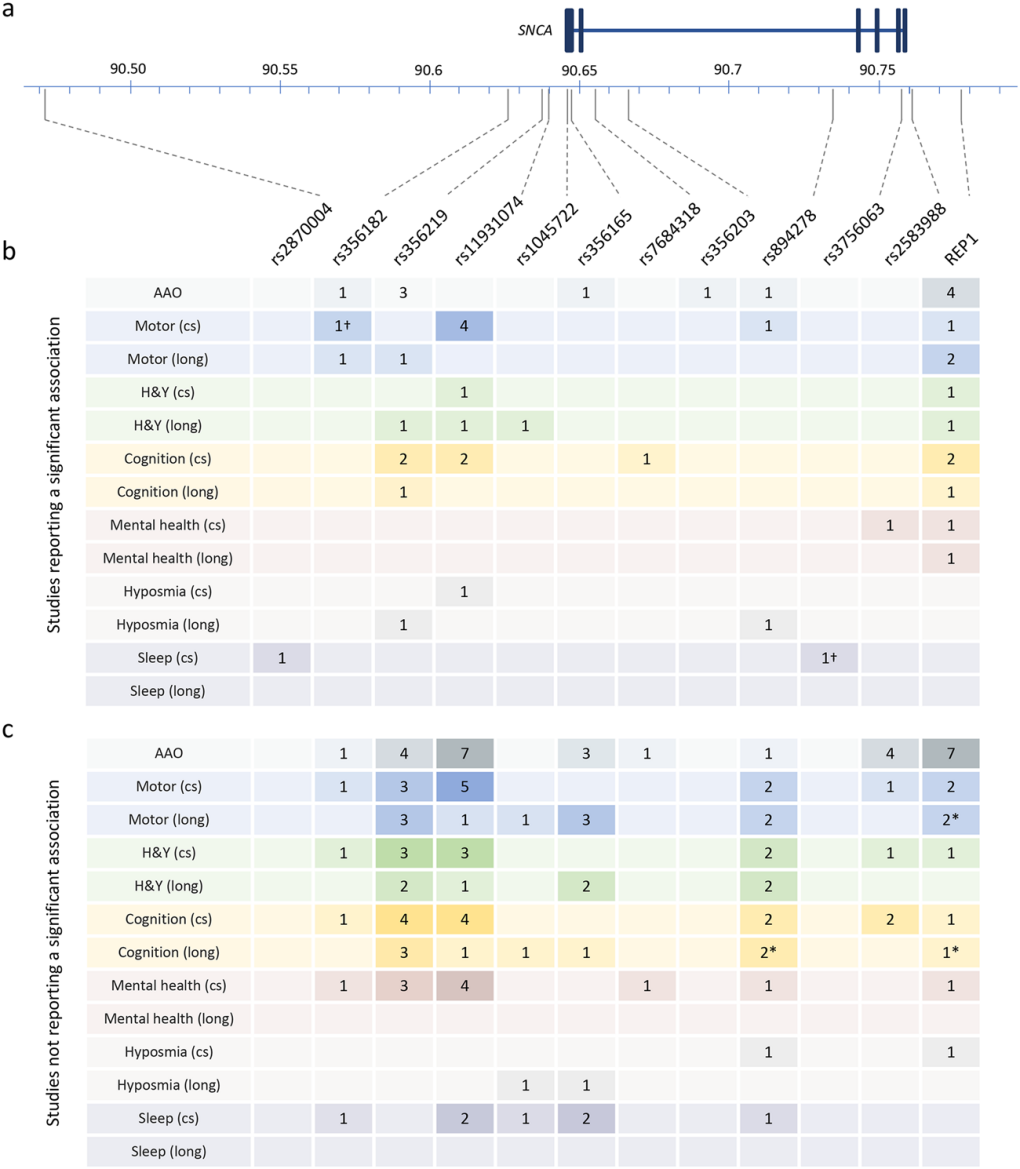
Summary of *SNCA* polymorphisms identified in this review that were reported to be associated with a clinical outcome in Parkinson’s disease. **a** Schematic of *SNCA* showing the location of each polymorphism. Exons are indicated by boxes. Chromosome 4 positions according to GRCh 37. Adapted from the output generated by LDLink^83^. **b** The number of studies reporting a significant association of a clinical outcome with the polymorphism indicated. **c** The number of studies reporting no significant association between a clinical outcome with the polymorphism indicated. In both **b** and **c**, the analysis of clinical outcomes is separated according to a cross-sectional (cs) or longitudinal (long) study design. A single study can be recorded more than once if both designs were included in the manuscript or if the study reported both significant and non-significant associations for a SNP in the same category. Full details of the SNPs and the outcomes in each category are given in Supplementary Tables 2 and 3. Asterisk (*): the unadjusted *p* value was <0.05 but the authors reported that the association did not remain significant after adjustment for multiple testing or covariates. Dagger (†): the study identified the association in a discovery cohort and replicated the finding in an independent cohort. AAO age at onset, cs cross-sectional, H&Y Hoehn and Yahr, long longitudinal.

A total of 51 different *SNCA* genetic polymorphisms were investigated, comprising 50 single-nucleotide polymorphisms (SNPs) and variations in the complex microsatellite D4S3481 known as REP1, located approximately 10 kb upstream of the translational start of *SNCA*. The location and population frequency (European and East Asian) of each polymorphism is provided (Supplementary Table 2). Of the 51 polymorphisms studied, 6 were reported to be significantly associated with AAO and 10 were reported to be significantly associated with a clinical outcome (Fig. 2 and Supplementary Table 3), defined by a *p* value of <0.05, unless the authors of the study specifically reported that the association was not significant after adjustment for multiple testing or covariates. A summary of all of the SNPs investigated and not shown to be significantly associated with any clinical outcome analyzed is given in Supplementary Fig. 1 and Supplementary Table 3.

### The association of SNCA polymorphisms with age at PD onset

One of the distinguishing features of autosomal-dominant PD caused by several *SNCA* gene mutations or multiplications is an early AAO, and this has led to an extensive investigation of common *SNCA* polymorphisms as modifiers of AAO in idiopathic PD. We identified 24 studies that assessed the role of *SNCA* polymorphisms on modifying AAO and 6 out of the 40 studied SNPs were associated with differences (Fig. 2 and Supplementary Table 3).

Seven small studies, including patients of European (*n* = 6)^11–16^ and Asian (*n* = 2)^16,17^ ancestry, did not find evidence that the *SNCA* REP1-risk genotype impacts AAO in PD (Fig. 2 and Supplementary Table 3). In contrast, three studies showed a significant association between REP1 and an earlier AAO^18–20^. Further, a meta-analysis on data from 4190 cases from 15 sites involved in the Genetic Epidemiology of Parkinson’s Disease (GEO-PD) Consortium, including some of the previously cited studies, found higher REP1 scores (defined here as the sum of allele score, with each 259-, 261-, or 263-bp allele contributing 0, 1, or 2 points, respectively) to be associated with an earlier AAO (hazard ratio (HR) = 1.06; *p* = 0.01)^21^.

Five studies took a broader approach to identify genetic AAO modifiers by analyzing the association of SNPs across many genes previously associated with the risk of sporadic PD, including polymorphisms in the *SNCA* region^10,11,22–24^. In a German study of 21 SNPs associated with PD risk, AAO was assessed in 1396 patients with PD and rs356219 from the *SNCA* region was shown to significantly contribute to earlier AAO (rs356219-G, *p* = 0.001)^23^. These data are consistent with reports from both a Chinese population of 685 cases (rs356219-G dominant model, *p* = 0.02)^25^ and a Spanish population of 898 cases (rs356219-G log-additive model, *p* = 0.0034)^24^, each of which reported that carriers of the rs356219-G allele had a significantly younger AAO than carriers of rs356219-A allele. Notably, the results from the Spanish population were extended to identify a three-loci epistatic combination of rs356219 with *RPTOR* rs11868112 and *RPS6KA2* rs6456121 that was associated with AAO in both the discovery cohort (*n* = 898; odds ratio (OR) 2.89; *p* < 0.0001) and a replication sample from the International Parkinson Disease Genomics Consortium (IPDGC; *n* = 4170; OR 1.56; *p* = 0.046–0.047). The most recent AAO study was also the largest study to be identified in this review, achieved by combining cases from the IPDGC with participants from the 23andMe research data set, which consisted of customers of the personal genetics company (23andMe, Inc.) who self-reported diagnosis and their age of PD diagnosis^10^: Blauwendraat et al. took two approaches, first performing a GWAS for AAO^10^. The top SNP associated with PD AAO in this GWAS was rs356203, resulting in an earlier AAO by approximately 0.6 years (*p_meta* = 1.90E−12). Interestingly, rs356203 has previously been shown to be associated with an increased risk of PD^8^ but has not been investigated for an association with other clinical outcomes in PD. Second, the authors analyzed an updated set of 44 PD-risk SNPs and identified variants in six different genes to be significantly associated with AAO, including the *SNCA* SNP rs365182 (*p* = 1.12 × 10^9^)^10^.

### Motor outcomes in PD

PD is primarily a movement disorder and accordingly motor signs and symptoms were the most frequent clinical outcomes evaluated. Of the 22 studies analyzing a total of 16 polymorphisms, 9 reported a significant association between a motor-related outcome and 4 SNPs^26–31^ or the REP1 polymorphism^32–34^ (Fig. 2 and Supplementary Table 3).

In cross-sectional analyses, significant associations of *SNCA* polymorphisms with Unified Parkinson’s Disease Rating Scale (UPDRS) scores were found in two Asian populations for REP1–263 allele carriers (UPDRS III, *β* 3.921; *p* = 0.026)^34^ and rs11931074 (UPDRS II, standardized coefficient (SC) −0.083; *p* = 0.035; UPDRS III, SC −0.140; *p* ≤ 0.001)^28^. However, these results were not replicated by other studies (Fig. 2 and Supplementary Table 3)^27,35,36^, including a large study from the Chinese National Consortium on Neurodegenerative Diseases (CNCPD; *n* = 2011)^36^. Cooper et al. showed an association between rs356182 and tremor-dominant (TD) vs. postural-instability gait disorder motor subtypes (corrected *p* = 0.04), which they subsequently replicated in an independent multi-center cohort (*p* = 0.002). In both the discovery and replication cohorts, the authors found that the rs356182-GG genotype was associated with a more TD phenotype^29^. Cooper et al. built on these findings and showed that rs356182 also predicted slower progression of motor impairment, with the annual rate of increase in UPDRS III scores found to be approximately 1 point per year less in rs356182-GG carriers compared to those with the AG or AA genotypes (*p* = 0.01)^29^. Further evidence that *SNCA* polymorphisms predict the rate of motor symptom decline in PD was reported in work from the Parkinson Environment and Genes cohort, a prospective, population-based study of patients in California, USA. In this study, the risk of faster decline of motor function (defined by a change of >5 points per year in UPDRS III score) was increased fourfold in carriers of the REP1–263 allele (OR 4.03; *p* = 0.004)^32^.

Few studies have addressed a possible association between *SNCA* polymorphisms and the development of motor complications related to dopaminergic treatment (Supplementary Table 3) and replication studies are lacking. The development of motor fluctuations and levodopa-induced dyskinesias (LIDs) up to 13 years from diagnosis was evaluated in patients from the prospective population-based Cambridgeshire Parkinson’s Incidence from GP to Neurologist (CamPaIGN) cohort. Carriers of rs356219-A were at increased risk of developing motor fluctuations compared to the rs356219-GG genotype (adjusted HR 1.902; *p* = 0.039), while no difference was observed in the development of LIDs between the two groups (unadjusted *p* = 0.907)^26^. Two studies assessed wearing off (WO): in an Italian study, carriers of the REP-263 allele were found to have a higher risk of WO compared to noncarriers at 10 years from disease onset (65.33% vs. 53.02%; log-rank test *p* = 0.028), although this result was not significant in multivariate Cox regression analysis^18^. In a separate Chinese study, rs11931074 was not found to be associated with WO (*p* = 0.520) in 724 patients with a comparably short disease duration (rs11931074-T carriers 4.61 ± 4.42 years; rs11931074-GG carriers 4.78 ± 3.97 years)^28^. Given the higher risk of motor problems with increasing disease duration, additional studies of more advanced PD will be needed to validate these results.

### Measures of global PD severity and disability

The Hoehn and Yahr (H&Y) scale provides an overall assessment of severity of PD based on clinical features and functional disability and, since its introduction, has remained the most widely used scale to describe the general severity of PD. Fourteen studies including a total of 31 different polymorphisms used the H&Y scale to measure PD severity (Supplementary Table 3), but the means by which the scale was used varied widely. For example, Davis et al. report no significant association between rs356219 or rs11931074 and the *annual change in H&Y stage*^22^, while in other studies these same SNPs were shown to be significantly associated with either a longer *time to a 0.5-point increase in H&Y stage* (rs356219; HR 0.20; *p* = 0.005)^37^ or a longer *time to reach H&Y stage ≥3* (mild-to-moderate disability) (rs11931074; HR 0.43; *p* = 0.03)^31^. Similarly, REP1 score was reported to be associated with a longer *time to reach H&Y stage ≥4* (severe disability) (HR 0.87; *p* = 0.046)^38^, while reports on the association of the REP1 polymorphism with *H&Y stage* at the time of examination are conflicting^34,36^. Shi et al. (CNCPD, *n* = 2011) reported no associations with H&Y stage in an exploratory study of 9 genetic variants from 6 different genes that included REP1^36^, while, in a smaller study (*n* = 172), Ng et al., found an association between REP1–263 and H&Y stage at examination (*β* 0.231; *p* = 0.008)^34^. These studies also vary greatly in the rigor of their design, but variability in the application of the H&Y scale makes a direct comparison of the results particularly challenging.

### Cognitive impairment and the development of dementia in PD

Cognitive decline is an important aspect of PD as it brings a substantial additional burden for the patient, caregivers, and the healthcare system. In 8 of the 21 studies that assessed the association between 36 *SNCA* polymorphisms and cognition, 4 SNPs^27,31,35,37,39^ and the REP1 polymorphism^18,34,38^ were shown to be significantly associated with measures of cognitive impairment or the diagnosis of dementia (Fig. 2 and Supplementary Table 3). Among these, rs356219 was the most frequently studied SNP. In the most comprehensive assessment of cognitive performance and rs356219, Mata et al. analyzed global cognitive function using the Montreal Cognitive Assessment (MoCA), and memory; attention and executive function; language processing; and visuospatial skills in 1079 patients with PD of European descent and found no significant association between rs356219 and any outcome^40^. Two small studies did show a significant association of rs356219 with global cognitive impairment, with conflicting results: in a Chinese explorative study of 50 patients, the rs356219-G allele was significantly associated with an 18% decreased risk of cognitive decline (*p* = 0.006), measured by the time to a 1-point decrease on the MoCA scale^37^. Conversely, a Brazilian study of 105 patients found that both rs356219 heterozygotes (rs356219-GA; OR 4.74; *p* = 0.021) and homozygotes (rs356219-GG; OR 5.74; *p* = 0.014) had significantly increased risk of cognitive impairment as defined by an education-corrected Mini-Mental State Examination (MMSE) cut-off^35^. Given Mata’s findings and the small number of participants and events observed in the latter two studies, there is currently limited support for a role of rs356219 in modifying cognitive impairment.

The association of rs356219 and dementia in PD has been studied in three independent populations. The rs356219-G allele was found to be associated with an increased risk of PD dementia (PDD; *p* = 0.01) in a Chinese population of 291 patients^39^. In contrast, studies of two European populations of patients with PD from the UK (CamPaIGN)^41,42^ and Spain^43^ found no link between rs356219 and the risk or development of dementia. All four studies used clinical diagnostic criteria for PDD (Supplementary Table 3). The differences in findings could be due to type I errors or reflect variance in ethnic backgrounds or study design, such as the inclusion of incident patients vs. patients from specialist units.

One of the largest studies to explore the impact of *SNCA* polymorphisms on cognitive decline analyzed the role of REP1 and 19 additional haplotype-tagging SNPs in 922 PD patients from the Molecular Epidemiology of Parkinson’s Disease study. Participants were assessed by telephone interview, either directly with the cases (Modified Telephone Interview for Cognitive Status) or via proxy (Alzheimer’s Disease Dementia Screening Interview)^38^. The group of patients with higher REP1 scores (linked with highest risk of PD) were shown to have *reduced* risk of developing cognitive impairment (HR 0.81; *p* = 0.0017)^38^. However, this protective effect of REP1 on cognitive decline was not replicated by an Italian study of 426 PD patients which showed that carriers of the REP1–263 allele (similarly linked with PD risk) showed significantly *increased* risk of dementia compared to carriers of shorter alleles (HR 3.03; *p* < 0.001)^18^ or by a Singapore study which showed that REP1–263 allele carriers were associated with lower MMSE scores (*β* –1.46; *p* = 0.010)^34^. While these later results are more aligned with the predicted effect of REP1 on disease outcomes, it is difficult to directly compare the results because of the scales used to asses cognition and the handling of the REP1 genotype are different in all three studies.

### Other non-motor symptoms in PD

Anxiety and depression are two of the most common mental health symptoms that affect people with PD. Seven studies from China (*n* = 6) and Brazil (*n* = 1) looked at depression or anxiety and a total of 11 *SNCA* polymorphisms (Supplementary Table 3). Only two studies reported a significant association: first, in a small (*n* = 105) Brazilian population, the rs2583988-TT genotype was found to significantly reduce the risk of depression, defined using the Beck Depression Inventory (OR 0.21; *p* = 0.046)^35^, and second, a large study in China (*n* = 1047) found that a REP1 “risk allele” (in this study defined by the copy number of a CA repeat) was associated with a decreased risk for the presence of mild-to-marked depression defined using the Hamilton Rating Scale for depression (OR 0.54; *p* = 0.049)^44^. Neither of these findings has been replicated.

As PD progresses, many patients will develop psychotic symptoms, primarily hallucinations, which is considered an important clinical milestone in the course of PD. However, only two studies reported the association between *SNCA* and the incidence of psychosis in PD (Supplementary Table 3). The first study found no evidence for an association with the *SNCA* REP1 alleles and psychotic symptoms measured by item 2 of the UPDRS part I (“*benign hallucinations with insight retained*” or worse) in the multi-center NeuroGenetics Research Consortium^45^. In the second study, with patients recruited in Italy, REP1–263 allele carriers were shown to have a 2.69-fold higher risk of developing visual hallucinations over the course of disease (*p* = 0.001) recorded from retrospective review of clinical records^18^.

Sleep disorders, including excessive daytime sleepiness and rapid eye movement (REM) sleep behavior disorder (RBD), usually increase in frequency over the course of PD. RBD is a parasomnia strongly linked to synucleinopathies and characterized by the absence of muscle atonia during REM sleep^46^. In a study of 325 patients with PD from Norway^47^, possible RBD (pRBD) was assessed using the RBD screening questionnaire (history of dream enactment without polysomnographically confirmed REM sleep without atonia). pRBD was significantly associated with the *SNCA* SNP rs3756063 (OR 1.44; *p* = 0.018) but not rs356165 or rs2245801. This result was replicated for rs3756063 in 382 patients from the Parkinson’s Progression Markers Initiative (PPMI) cohort with marginal significance (OR 1.35; one-sided *p* = 0.036)^47^. Recently, a large study analyzed the effect of four other *SNCA* SNPs, selected for their association with risk of PD or isolated RBD, in the Norwegian and PPMI cohorts and an additional cohort from McGill University^48^. Meta-analysis of the three cohorts identified a significant association of rs287004 with a reduced incidence of pRBD in PD (OR = 0.76; *p* = 0.009)^48^.

Two of the studies identified in this systematic review^49,50^ diagnosed RBD using the “gold standard” set by the International Classification of Sleep Disorder-II criteria^51^. These studies did not identify an association of RBD with multiple *SNCA* genetic polymorphisms in Chinese^49^ or Italian^50^ populations (Supplementary Table 3), but given their relatively small size (*n* = 57^50^ and 152^49^), as typified by studies of clinically defined RBD using overnight polysomnography, larger studies will be needed to characterize the impact of *SNCA* polymorphisms in formally diagnosed RBD in PD.

Hyposmia, identified as reduced sensitivity to odor, is a common non-motor symptom of PD and can antedate the typical motor symptoms by several years^52^. However, only three small studies identified in this review have explored the association of *SNCA* polymorphisms with hyposmia, each using the 16-item odor identification “Sniffin’ Sticks” (SS-16) tool to measure hyposmia in PD. Of these, 1 Chinese study of 50 patients followed for 30 months suggests that of the six SNPs selected, rs894278-G (HR 0.47; *p* = 0.029) and rs356219-G (HR 0.32; *p* = 0.021) were associated with slower olfactory impairment^37^. Another Chinese study of 218 patients report that the rs11931074-TT genotype increased the risk of hyposmia more than 3-fold compared with the GG genotype (crude OR 3.41; *p* = 0.011) but did not find any significant associations for rs894278^53^. The third study (*n* = 295) found no associations for SS-16 outcome measures and REP1 in a population from the Netherlands (Supplementary Table 3)^54^.

## Discussion

In this systematic review, we provide a comprehensive summary of available data regarding the association of *SNCA* polymorphisms and clinical outcomes or AAO in patients with idiopathic PD. In total, 58 studies were identified that analyzed not only the association of 51 different *SNCA* polymorphisms with a broad range of outcomes, most commonly addressing AAO or motor impairment that is the core feature of PD, but also a broad range of non-motor symptoms, including cognitive impairment, sleep, mental health, and psychosis. Despite the large number of studies that have been conducted, the only associations that were replicated by independent studies were between *SNCA* polymorphisms and AAO, and no robust associations were identified for any of the clinical outcomes considered. This can not only be attributed to the lack of consistency in study designs and methodological shortcomings, but may also indicate that common *SNCA* variants are not major drivers of the heterogeneity in clinical presentation and progression that is observed in PD.

For the majority of polymorphisms (*n* = 39; 76%) identified in this review, no significant associations were reported with a clinical outcome or AAO, although it is notable that 31 of these SNPs were only included in 1 or 2 studies (Supplementary Fig. 1 and Supplementary Table 3). At the other extreme, the most frequently analyzed polymorphisms rs356219 and REP1 were each included in 21 studies. These two polymorphisms were among the very first genetic polymorphisms studied as modifiers of PD risk^16,55,56^ and are common in populations of both European and Asian ancestry, which may contribute to their popularity. The only replicated association for both rs356219 and REP1 was with AAO (rs356219, *n* = 3^23–25^; REP1, *n* = 4^18–21^). Clinical outcomes were included in 17 of the studies analyzing rs356219 and 11 of the studies analyzing REP1. Multiple significant associations, spanning the spectrum of the clinical outcomes identified in this review, were reported for both rs356219 and REP1, but notably none were replicated in independent populations (Fig. 2 and Supplementary Table 3). Therefore, the results warrant cautious interpretation. The possible reasons for the lack of robust associations are discussed below.

While the inclusion criteria pre-specified idiopathic PD diagnosed using defined diagnostic criteria, no limitations were put on other aspects of the study design, and the review revealed a striking heterogeneity in the design of the study cohorts, sample size, and the choice of the *SNCA* polymorphisms and the outcome measures, each of which can diminish the capacity to compare the effects of *SNCA* variants among the different studies. Most striking was the large heterogeneity in the outcome measures used to assess the severity of symptoms and disease progression, with >30 different clinical scales or questionnaires identified in the review, many of which were assessed only once. Adding to the heterogeneity, even when studies used the most common PD rating scales, the way in which these scales were applied varied greatly. This was not only exemplified in the analysis of H&Y staging (see “Results”) but was also notable in the application of the UPDRS and MMSE (see outcomes in Supplementary Table 3). There were also considerable differences in the demographic and clinical characteristics of the populations studied. Most were recruited from hospitals or specialized clinics and very few were population-based cohorts, which can limit the generalizability of the results. Further, most cohorts were limited to Caucasian or Asian populations, and ethnic differences can preclude the generalization of the results in genetic studies.

Increasing evidence suggests that variants in *SNCA* modify the risk for developing PD by altering the levels of α-synuclein and promoting the abnormal aggregation into LBs. In this model, small changes in α-synuclein expression may, over many decades, predispose to PD and different *SNCA* variants can contribute to this by altering the transcriptional regulation of α-synuclein expression. Of the polymorphisms identified in this review, the most compelling evidence for a mechanistic role in α-synuclein regulation is found for REP1 and rs356219^57–60^. These common genetic variants have been correlated with higher α-synuclein expression in vitro^58^ or with elevated brain or peripheral levels of α-synuclein in vivo^57,59^ and it is a biologically plausible hypothesis that these same mechanisms could impact the heterogeneity that is observed in PD. The results of this review support a role for these variants in modifying AAO; however, we did not find any strong evidence for a link to disease outcomes. Indeed, some studies even suggest the opposite findings, with those SNPs increasing PD risk being linked to fewer or milder symptoms, with examples found for REP1^38^, rs356219^26^, rs11931074^30,39^, and rs356182^29^. These observations could reflect the shortage of well-designed replication studies but may also indicate that *SNCA* is an initiator of PD risk but not of the development of specific symptoms or changes in the rates of disease progression. This hypothesis is in line with a recent study combining data from 13 longitudinal PD cohorts, which found that a genetic risk score based on 31 PD-risk SNPs was inversely associated with AAO but not with a comprehensive battery of clinical outcomes^61^.

Several limitations must be considered in interpreting the results presented in this review. Despite the large number of studies identified, the heterogeneity in study design meant that it was not possible to compare the effect sizes among the studies or perform a meta-analysis. Further, we only reported the effects of multiple testing if the authors themselves included this as part of their analysis plans, although the assessment of potential sources of bias served to highlight some studies in which this was a weakness in the design (Supplementary Table 1). The review inclusion criteria may have missed some works, especially those that included negative findings related to *SNCA* as data not shown, and this highlights the plausible existence of positive reporting bias. Finally, the Preferred Reporting Items for Systematic reviews and Meta-Analyses (PRISMA) statement was used as a guide to ensure a rigorous review process; however, we note that the protocol for our review was not published before the search was initiated. The strengths of our review include the broad inclusion criteria, which captured studies of any clinical outcome associated with PD and including any *SNCA* polymorphism. Furthermore, several aspects of the design of each study were evaluated using pre-determined criteria in order to provide an assessment of the sources of bias in the studies.

To clarify the role of genetic variation in the *SNCA* region in PD heterogeneity, further studies are required. This review has highlighted the need for well-designed studies with standardized cohort designs, optimally recruiting patients early in the disease and following them prospectively using comprehensive batteries of clinical scales to measure disease severity and progression. Further, approaches to harmonize how disease progression is measured should be prioritized. One explanation for the broad range of tools used is that we currently lack clinical tools that assess PD progression in an accurate and meaningful way across the spectrum of disease stages. A new approach was recently adopted in a GWAS of cognitive and motor progression in which multiple assessments were combined in a data-driven principal components analysis to derive scores for composite, motor, and cognitive progression^62^. Alternatively, biomarkers may prove to be more sensitive measures of disease progression, with less susceptibility to the effects of subjectivity, medication, and placebo. Clearly, further efforts to improve phenotypic measures and define disease progression should be pursued.

There was a notable scarcity of population-based incident cases representative of the general PD population, which may lead to bias if the associations between polymorphisms and clinical features vary by age. The mean age of PD onset of the study cohorts reported in the studies was 58.8 (±5.3) years and the mean age at examination was 63.7 (±4.5) years. This is typical of general research studies, which are generally unrepresentative of the population age distribution of PD^63^. Finally, it is plausible that the effect sizes of *SNCA* polymorphisms will be small, and therefore large studies are needed to detect small effect sizes, as was demonstrated by the AAO GWAS of Blauwendraat et al.^10^. This can result in a trade-off between sample size and data quality, but there is an increasing drive for world-leading PD cohorts to collaborate or for journals to require the deposition of original data in public databases, and these initiatives will provide excellent platforms to further probe the role of *SNCA* variants in PD progression.

The lack of robust findings casts doubts on the relevance of *SNCA* SNPs in modifying disease course in PD and suggests that the genetics of PD risk and progression are largely separate. However, methodological aspects may explain some of the discrepancies in the effects of polymorphisms across the different studies. Most significant associations identified in this review found that *SNCA* polymorphisms had minor effects on the clinical outcomes and so, even if validated, these findings are not currently relevant in a clinical setting. However, given that α-synuclein is a major drug target, they have the potential to be used as a genetic risk stratification tool to reduce variability in clinical trials, and so further studies using large, well-designed cohorts are warranted.

## Methods

### Systematic review strategy

The review process was conducted according to the PRISMA statement^64^. A systematic review of literature was performed to summarize all available primary research reporting the association of *SNCA* polymorphisms with clinical features of PD. The following inclusion criteria were used: subjects were patients with PD diagnosed according to defined diagnostic criteria (i.e., UKBB, Gelb, Bower or other clearly defined criteria that were endorsed by a movement disorder expert (G.A.)), genetic polymorphisms were within the *SNCA* gene region, outcomes were any clinical feature attributed to PD or AAO, the association of *SNCA* polymorphisms with outcomes was assessed statistically, and the source was primary literature in English. Full exclusion criteria are listed in Supplementary Table 4.

The searches were performed on June 15, 2020 by the first and last author using two databases (PubMed and Web of Science) and the following search terms: Parkinson* AND *SNCA* AND (variant* OR polymorphism* OR SNP* OR GWAS OR genome-wide) (Supplementary Table 5). Five hundred and ninety-seven unique publications were identified, of which 431 were excluded for clearly not meeting the inclusion and exclusion criteria based on reading the title and abstract (Supplementary Fig. 2). The remaining 166 publications were read in full by the first and last author. Cases of diverging opinion about an article’s eligibility based on the inclusion/exclusion criteria were discussed at both stages. Fifty publications were found to be eligible for this review (Fig. 1). Eight further articles were retrieved from a manual search of the 50 evaluated papers’ references (*n* = 5) and from the wider PD literature (*n* = 3) (identified in Table 1). For the final 58 publications, the publication year, country of study, cohort name or main study site, criteria for diagnosis of PD, number of patients, AAO, age at examination, disease duration, number of patients followed longitudinally, length of follow-up, *SNCA* polymorphisms, clinical features analyzed, and result of association analyses (*p* value of best model) were retrieved, as available. An overview of the papers included in this review (Table 1) and of the *SNCA* polymorphisms and outcomes assessed (Supplementary Tables 2 and 3) are provided. The risk of bias for each study was assessed by the first and last author using a simple traffic light system to grade potential sources of bias as low level (green), of some concern (yellow), or high level (red). A third author (J.L.) resolved cases of diverging assessment. The factors taken into consideration were sample representativeness, adequate PD definition, adequate assessment of outcomes, multiple comparisons tests, pre-specified plan, and replication in an independent cohort (Supplementary Table 1).

### Reporting summary

Further information on research design is available in the Nature Research Reporting Summary linked to this article.

## Supplementary information

Supplementary Information

Supplementary Data 1

Reporting Summary

## Data Availability

The data that support the findings of this systematic review are provided in the Supplementary files.
