# Supplementary material for: A systematic review of associations between common SNCA variants and clinical heterogeneity in Parkinson’s disease
Source: NPJ Parkinsons Dis. 2021 Jul 1;7:54. doi: 10.1038/s41531-021-00196-5 (PMC8249472; doi:10.1038/s41531-021-00196-5)
Supplement: Supplementary file 1 — Supplementary Information [file 41531_2021_196_MOESM1_ESM.pdf]

**Supplementary information for a systematic review of associations between common *SNCA* variants and clinical heterogeneity in Parkinson's disease**

*Supplementary excel files*

**Supplementary Table 1**. Risk of bias assessment for the articles included in the review

**Supplementary Table 2**. Summary of the polymorphisms identified in the review

**Supplementary Table 3**. Summary of the outcomes and polymorphisms reported in each article included in the review

**Supplementary Table 4.** Exclusion criteria used to screen studies from the database search.

| **Exclusion criteria** |
|---|
| Not primary literature |
| Not Parkinson's disease |
| Only LRKK2-associated Parkinson's disease |
| Clear diagnostic criteria not reported |
| Genetic polymorphisms not in *SNCA* gene region |
| Genetic variants other than individual polymorphisms considered, e.g. haplotypes, mutations or copy number variations |
| Only the association with risk of developing Parkinson's disease reported |
| Only the association with neuroimaging markers reported |
| Subgroups of patients with Parkinson's disease only compared to healthy controls |
| The study was only descriptive |
| In a language other than English |

**Supplementary Table 5.** Search terms used to identify studies related to the objective of the systematic review.

| | **PubMed** | **Web of science** |
|---|---|---|
| Search terms | (parkinson*) AND (SNCA) AND (variant* OR polymorphism* OR SNP* OR GWAS OR genome-wide)<br><br>English filter on | **ALL FIELDS:** ((parkinson*) AND (SNCA) AND (variant* OR polymorphism* OR SNP* OR GWAS OR genome-wide))<br><br>**LANGUAGE:** (English)<br><br>**DOCUMENT TYPES:** (ARTICLE OR REVIEW)<br><br>**Timespan:** All years.<br><br>**Indexes:** SCI-EXPANDED, SSCI, A&HCI, ESCI. |
| Yield | 472 results | 451 results |

**Supplementary Figure 1.**

a

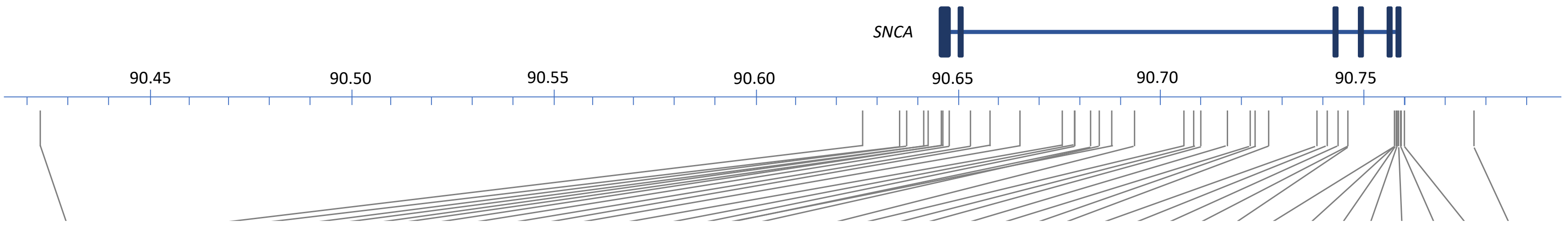


b

| | rs11732740 | rs356181 | rs181489 | rs356218 | rs356220 | rs356221 | rs3857053 | rs18618986 | rs17016074 | rs17180453 | rs3775423 | rs3822086 | rs3857059 | rs2736990 | rs2572324 | rs3910105 | rs10516845 | rs3796661 | rs356164 | rs356186 |
|---|---|---|---|---|---|---|---|---|---|---|---|---|---|---|---|---|---|---|---|---|
| Motor (cs) | | 1 | | | 1 | | | | | | | 1 | | 3 | | | | | | |
| Motor (long) | | | | | 1 | | 1 | | | | | 1 | 1 | 1 | | | | | | 1 |
| H&Y (cs) | | | | | | | | | | | | | | 2* | | | | | | |
| H&Y (long) | | | | 1 | 1 | | 1 | | | 1 | 1 | 1 | 1 | 2 | 1 | | | 1 | | 2 |
| Cognition (cs) | | 2 | | | 1 | | | | | | | 1 | | 2 | | 1 | | | | |
| Cognition (long) | | | | 1 | | | 1 | | | 1 | 1 | | | 1 | 1 | | | 1 | | 2 |
| Mental health (cs) | | | | | 1 | | | | | | | 1 | | 2 | | | | | | |
| Mental health (long) | | | | | | | | | | | | | | | | | | | | |
| Hyposmia (cs) | | | | | | | | | | | | | | | | | | | | |
| Hyposmia (long) | | | | | | | 1 | | | | | | | | | | | | | 1 |
| Sleep (cs) | 1 | | | | | | 1 | 1 | 1 | | | | | | | | | | | |
| Sleep (long) | | | | | | | | | | | | | | | | | | | | |
| AAO | | | 3 | 1 | 1 | 2 | | | | 1 | 1 | 2 | 1 | 3 | 1 | | 2 | 1 | 2 | 3 |

| | rs3775438 | rs3775439 | rs9995651 | rs2583959 | rs6848726 | rs3775444 | rs10155475 | rs2737020 | rs10005233 | rs2737012 | rs6532191 | rs3756059 | rs1372520 | rs2245801 | rs2301135 | rs2301134 | rs2619363 | rs2619364 | rs1372525 |
|---|---|---|---|---|---|---|---|---|---|---|---|---|---|---|---|---|---|---|---|
| Motor (cs) | | | | | | 1* | | | | | | | | | | | | | |
| Motor (long) | | | | | | | | | | | | | | | | | | | |
| H&Y (cs) | | | | | | | | | | | | | | | | | | | |
| H&Y (long) | 1 | 1 | 1 | 1 | | | 1 | | 1 | 1 | 1 | 1 | 1 | | 1 | | 1 | | |
| Cognition (cs) | | | | | | 1 | | | | | | | | | 1 | | 1 | 1 | 1 |
| Cognition (long) | 1 | 1 | 1 | 1 | | | 1 | | 1 | 1 | 1 | 1 | 1 | | 1 | | 1 | | |
| Mental health (cs) | | | | | | 1 | | | | | | | | | | | | | |
| Mental health (long) | | | | | | | | | | | | | | | | | | | |
| Hyposmia (cs) | | | | | | | | | | | | | | | | | | | |
| Hyposmia (long) | | | | | | | | | | | | | | | | | | | |
| Sleep (cs) | | | | | | | | | 1 | | | | | 1 | | | | | |
| Sleep (long) | | | | | | | | | | | | | | | | | | | |
| AAO | 1 | 1 | 1 | 1 | 2 | 1 | 1 | 2 | 1 | 1 | 1 | 1 | 1* | | 1 | 2 | 3 | 2 | |

**Supplementary Figure 1.** Summary of *SNCA* polymorphisms identified in this review that were reported not to be associated with clinical outcome measures or age of onset.

(a) Schematic of SNCA showing the location of each polymorphism. Exons indicated by boxes. Chromosome 4 positions according to GRCh 37. Adapted from the output generated by LDLink.[1] (b) The number of studies reporting no significant association between a clinical outcome with the polymorphism indicated. The analysis of clinical outcomes is separated according a cross-sectional or longitudinal study design, and accordingly, a study can be recorded more than once if both designs were included in the manuscript. For full details of the studies are given in Supplementary file 3 and 4.

* The *p* value for the unadjusted association test was <0.05 but the authors reported it not to remain significant after adjustment for multiple testing or covariates.

References

1 Machiela, M. J. & Chanock, S. J. LDlink: a web-based application for exploring population-specific haplotype structure and linking correlated alleles of possible functional variants. *Bioinformatics* **31**, 3555-3557, doi:10.1093/bioinformatics/btv402 (2015).

**Supplementary Figure 2.**

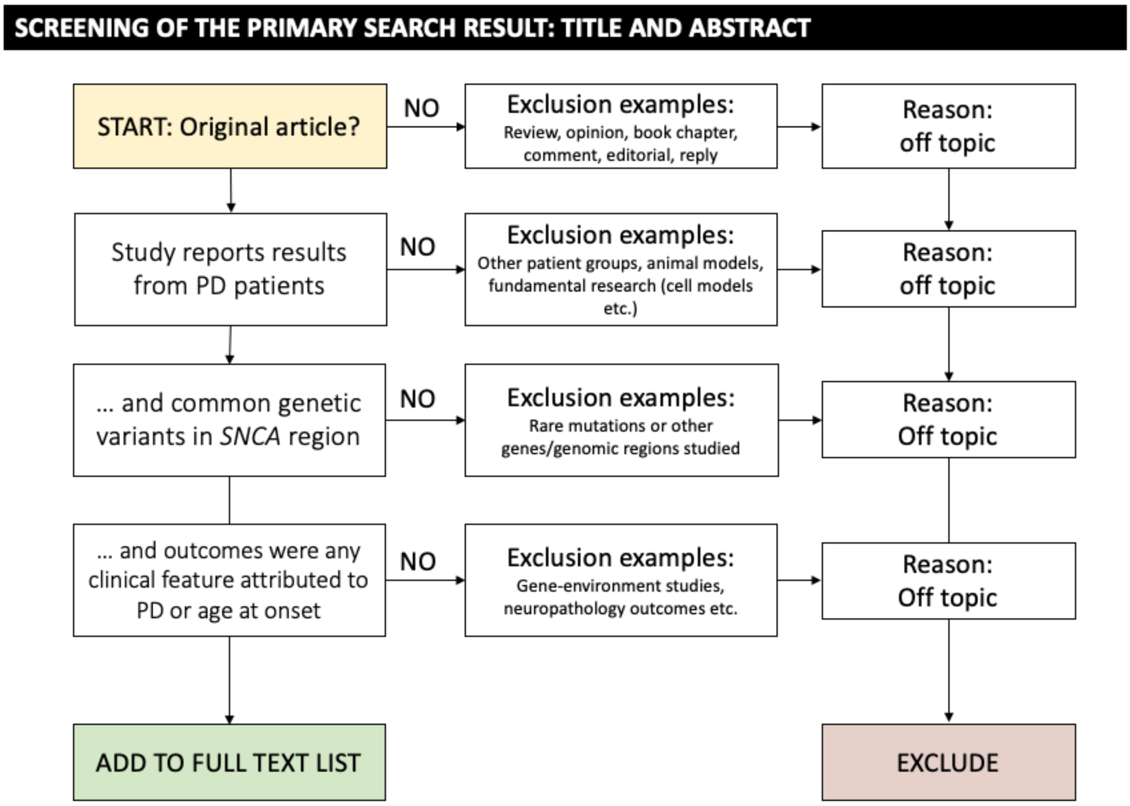


**Supplementary Figure 2.** Flow diagram for the first round of screening of the articles identified by the database searches. In the first round of filtering, articles were excluded for clearly not meeting the inclusion and exclusion based on the contents of the title and abstract. If the relevance was unclear from the title/abstract alone, the article proceeded to the next step of the screening process.
